# First person – Kayla Mills

**DOI:** 10.1242/bio.060102

**Published:** 2023-08-15

## Abstract

First Person is a series of interviews with the first authors of a selection of papers published in Biology Open, helping researchers promote themselves alongside their papers. Kayla Mills is first author on ‘ Low colostrum intake results in potential accumulation of peroxisome lipid substrates in vaginal tissues of 3-week-old gilts’, published in BiO. Kayla conducted the research described in this article while a graduate research assistant in Theresa Casey and Kara Stewart's lab at Purdue University, West Lafayette, IN, USA. She is now a research physiologist (USDA-ARS) at USDA, ARS, Beltsville Agricultural Research Center, Animal Biosciences & Biotechnology Laboratory, Beltsville, investigating the use of multiomic approaches to identify infertility biomarkers and further understand the mechanism behind infertility in livestock.


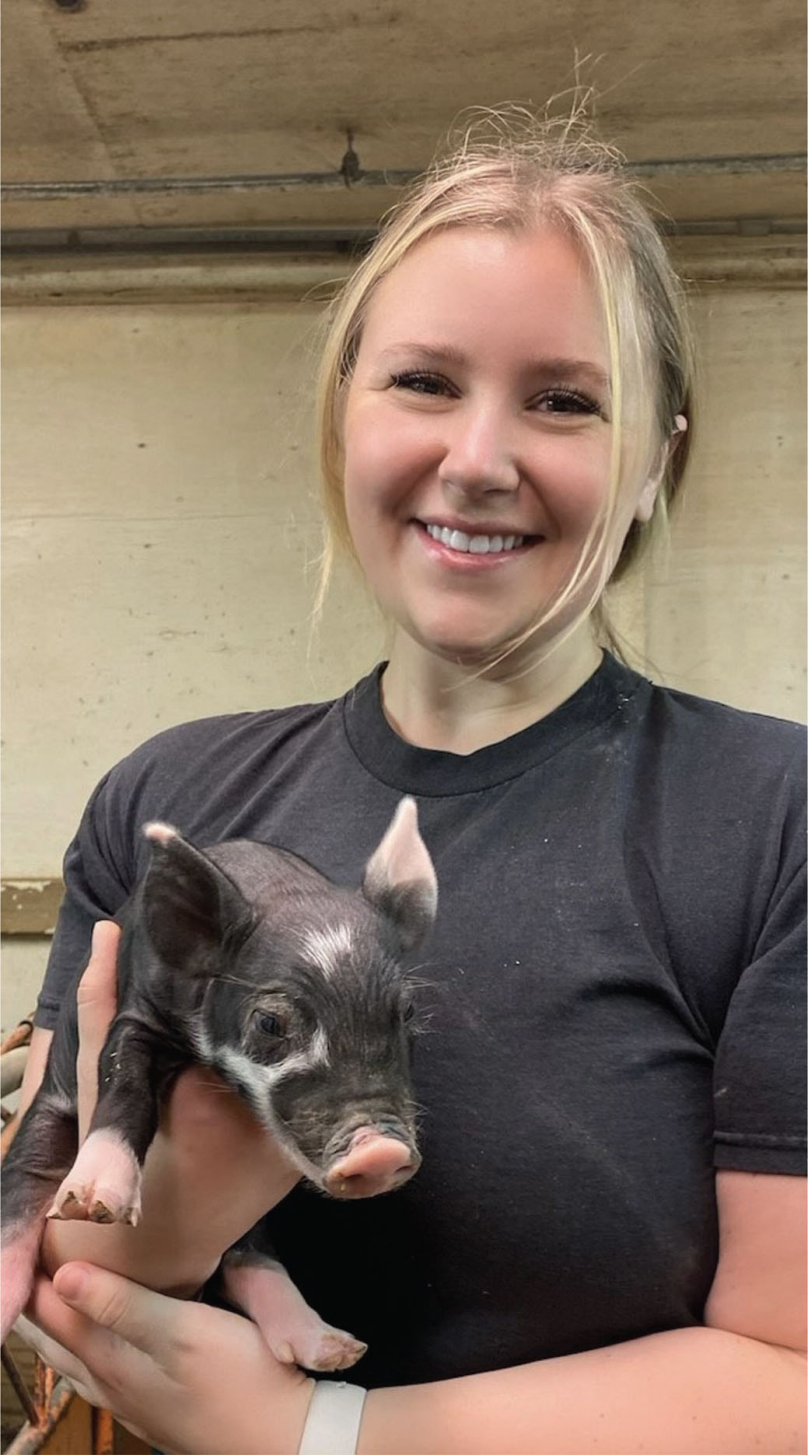


**Kayla Mills**

**Describe your scientific journey and your current research focus**

I began my scientific journey about a decade ago when I started working in a monogastric nutrition laboratory as an undergraduate research assistant. It was from there that I decided to pursue a master's in swine nutrition, but also maintained an interest in reproductive physiology as nutrition heavily influences fertility status. Following the completion of my master's I pursued a PhD in reproductive physiology with an emphasis on the identification of fertility biomarkers in swine. During my doctoral program, I became familiar with several omic approaches and how these tools can help better our understanding of complex traits such as fertility. In 2021, I joined the Animal Biosciences & Biotechnology Laboratory in Beltsville, MD, as a research physiologist with the USDA. My current research program is focused on the development of new technologies and methods to enhance the fertility, utilization, and long-term storage of swine germplasm. Here, I am utilizing a multiomic approach to understand the physiology behind subfertility and effects of cryopreservation on the sperm cell to construct low-density assays for biomarker-assisted fertility and cryotolerance assessment. The goal of this research is to provide the swine and other applicable livestock industries with the ability to propagate genetic stocks for efficient meat production and define the impact of genetics on cryopreservation success and government institutions currently maintaining heritage, research, and other ‘at-risk’ lines to preserve genetic diversity.

**Who or what inspired you to become a scientist?**

I originally wanted to become a veterinarian but my graduate mentor, Dr Caitlin Vonderohe, convinced me to give research in animal science a try. She was a PhD student in the monogastric laboratory that hired me as an undergraduate research assistant. The project I assisted her with evaluated the impact of rearing swine in an antibiotic-free setting on the environment. I became so inspired by the work being done at Purdue University that I decided to switch career paths. I enjoyed knowing that the research I was contributing to was helping both animals and people.

I enjoyed knowing that the research I was contributing to was helping both animals and people.

**How would you explain the main finding of your paper?**

Colostrum is the first milk available to newborns and is a concentrated source of nutrients, essential fatty acids, and other important factors essential for proper development. It has also been documented that the level of colostrum intake is related to long-term success within the swine breeding herd. Thus, identifying a way to detect young female swine (gilts) that drink adequate amounts of colostrum would help in the selection of breeding herd replacements. In our previous work, the vaginal lipidome (cellular fat composition) of gilts on day 2 of life was reflective of colostrum consumption and the vaginal lipidome in gilts at weaning (21 days of age) were related to long-term fertility. Our aim was to determine if differences in vaginal lipidome profiles of gilts at weaning were reflective of variation in colostrum intake. Here, we evaluated traditional markers of colostrum intake (immunocrit ratio and 24 h weight gain) and their relationship to vaginal lipidome profiles of gilts at weaning. Gilts that had high versus low immunocrit ratios and 24 h weight gain showed a difference in vaginal lipid profiles and these lipids may serve as excellent biomarkers of colostrum intake. We also observed that gilts with low colostrum intake shared a similar lipid profile to the infertile gilts identified in our previous study. Therefore, colostrum intake of gilts during the first 24 h of life is crucial to their longevity within the sow herd, and thus warrants future research in the proper management colostrum intake during the first 24 h of life.

…colostrum intake of gilts during the first 24 h of life is crucial to their longevity within the sow herd…

**What are the potential implications of this finding for your field of research?**

Currently, sow removal rates are at an all-time high with the number one reason being reproductive failure. In addition, about half of the females produced to replace the sows being removed are not exhibiting estrus and the reason for this is still unknown. The findings in this paper highlight the importance of perinatal nutrition and its relationship to reproductive tract development. The ability to increase sow longevity within the breeding herd through proper colostrum feeding and selection strategies would be highly advantageous to the swine industry as this would improve overall efficiency on-farm and reduce the need to produce so many replacement animals.

**Figure:**
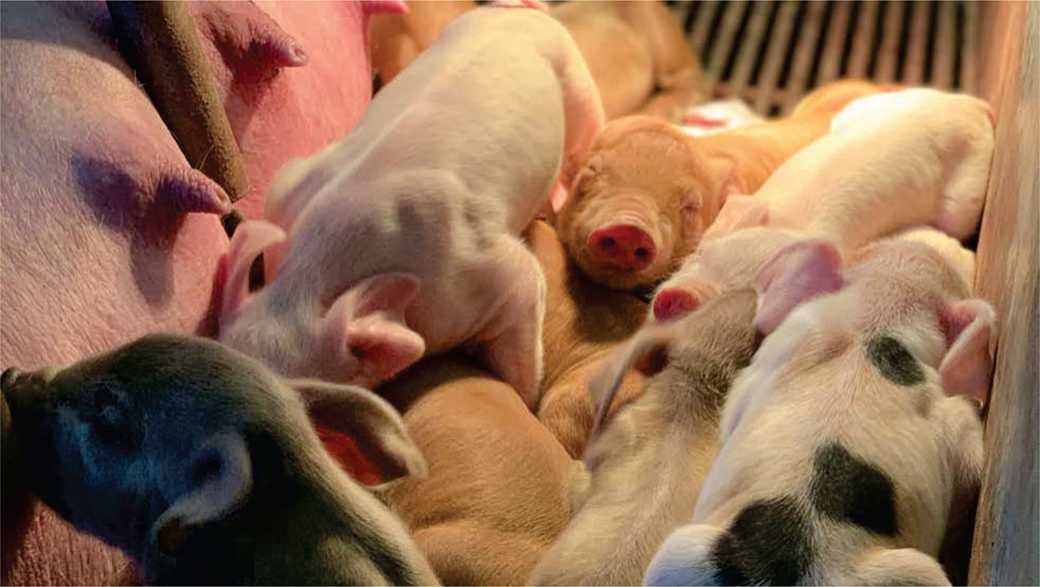
**Piglets resting shortly after they are born while a littermate is suckling.** This 24-h window of opportunity is crucial for proper reproductive tract development.

**Which part of this research project was the most rewarding?**

The most rewarding aspect of this project was validating that colostrum intake is what dictates the vaginal lipidome profile seen in infertile gilts, thus giving us hope that the fertility issues that are seen on-farm could likely be improved through proper management practices and it is not too late to fix.

**What do you enjoy most about being an early-career researcher?**

I really enjoy the aspect that this only the beginning. I am so excited to see what comes of my research throughout the rest of my career.

**What piece of advice would you give to the next generation of researchers?**

Never limit yourself based on the opinion of others. If you're willing to work hard enough for something, go for it!

**What's next for you?**

In the coming years, I will continue to build my research program here in Beltsville and aim to create low-density assays for biomarker-assisted selection to help farmers and the swine industry.
